# Supplementary material for: Conditional chemoconnectomics (cCCTomics) as a strategy for efficient and conditional targeting of chemical transmission
Source: eLife. 2024 Apr 30;12:RP91927. doi: 10.7554/eLife.91927 (PMC11060718; doi:10.7554/eLife.91927)
Supplement: Figure 3—source data 3. [file elife-91927-fig3-data3.zip › Figure 3 source data3 part 1.zip!/Figure 3-Source data 3-part1/1071f3-1-CRISPResso_Report_jvi77nmx.zip!/CRISPResso_on_1071f3_2/1c.Alignment_barplot.pdf]

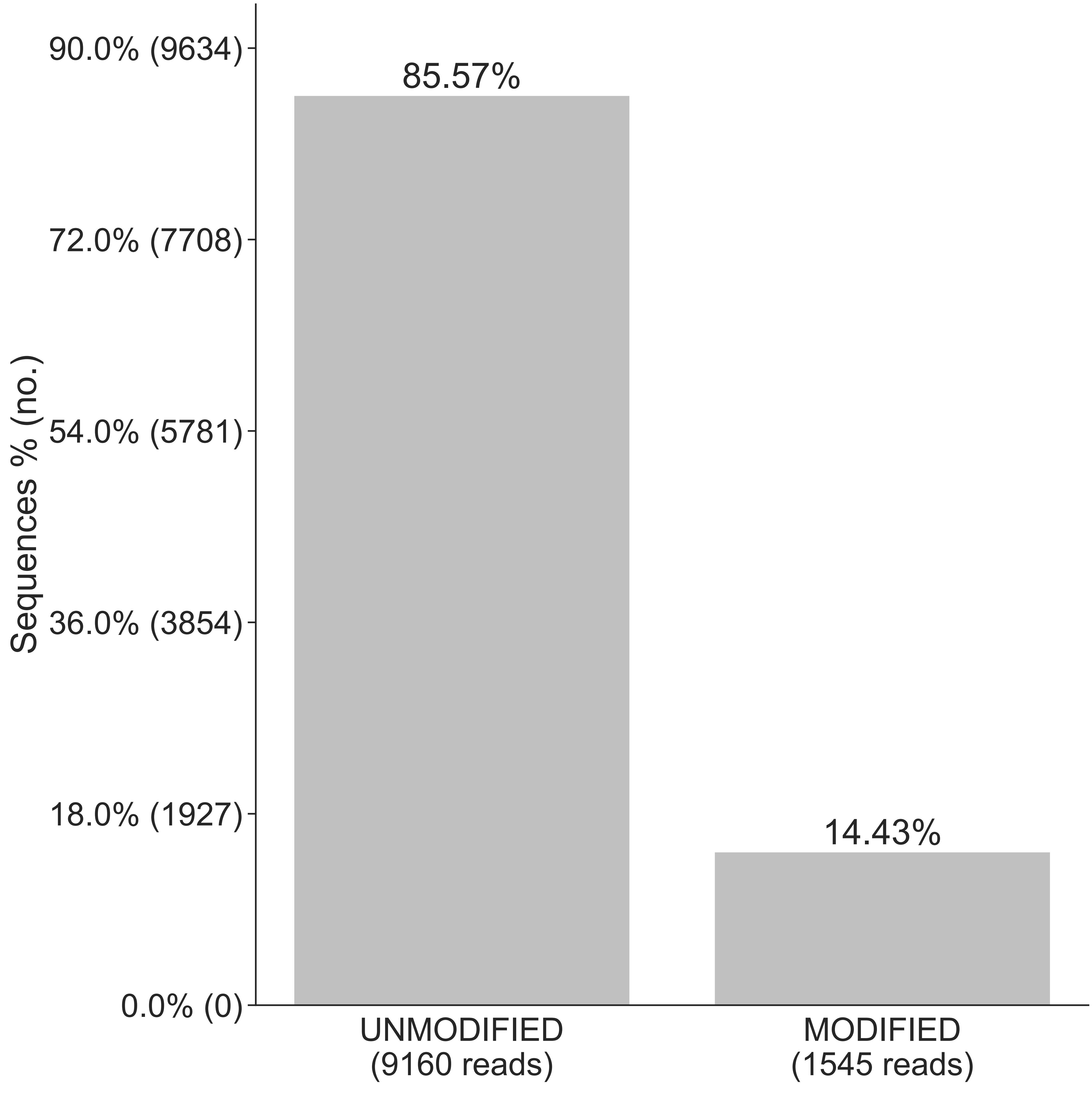
Sequences % (no.)
90.0% (9634)
72.0% (7708)
54.0% (5781)
36.0% (3854)
18.0% (1927)
0.0% (0)
85.57%
14.43%
UNMODIFIED
(9160 reads)
MODIFIED
(1545 reads)
